# Hospital Meetings, &c.

**Published:** 1900-06-30

**Authors:** 


### HOSPITAL MEETINGS, &C.

### NATIONAL SOCIETY FOR EMPLOYMENT OF EPILEPTICS.

The annual meeting of the Governors of this society was held on Monday, June 11th, at 12, Buckingham Street, Strand. The chair was taken by Mr. E. Montefiore Micholls, and the reports of the hon. medical staff and of the Executive Committee for the year 1899 were received and adopted. In the first-named report it is stated that amongst the epileptic "Colonists" at Chalfont a marked improvement has been noted in the majority of instances both as regards the general state of health and the disease, and that the progress has been especially noticeable in the younger cases of both sexes, the improvement both in their general bodily development, and more particularly in their disease being highly satisfactory. The report of the Executive Committee states: There are many thousands of epileptics who stand in urgent and dire need of such provision as the colony affords, and until every one of these is provided for the ultimate goal will not have been attained. The report contains an extract from an appeal issued by the Duke of York, as president of the society, in which His Royal Highness observes: "Of all the more serious forms of natural affliction epilepsy is probably the most prevalent. It would seem, therefore, that it should appeal to almost universal sympathy, yet hitherto the supporters of the National Society have been distinguished rather by their individual generosity than by their numerical importance. For its permanent stability it is much to be desired that the society should stand, as it were, upon a broader basis, and members of all classes are therefore, according to their respective means and opportunities, invited to participate in and support this very humane and necessary work."

### ROYAL ORTHOPÆDIC HOSPITAL.

The annual meeting of this institution was held on the 12th inst. at the hospital, Mr. H. H. Marks, M.P., presiding. There were also present: Mr. Ernest Flower, M.P., Major Ricarde-Seaver, Mr. H. A. Reeves, and Mr. T. S. Mansford (secretary). The annual report stated that on account of the necessity for improving the sanitary conditions of the hospital the building had to be closed for eight weeks last year, and an outlay of £2,000 was incurred, for which the committee appealed to the public. At the same time the committee gratefully acknowledged increased help in the way of subscriptions and donations, especially the grant of £250 from the Prince of Wales's Hospital Fund. The number of in-patients last year was 126, and out-patients 671, rather less than usual in each case on account of the closing of the hospital. The report was adopted, and the committee and officers re-elected. At the conclusion of the business a special court of governors was held, at which Mr. H H. Marks moved a resolution stating that the court, having considered the arrangements and negotiations for the disposal of the present site and the acquisition of a new one, approves and authorises the committee to carry the same into effect and to erect a new hospital. Major Ricarde-Seaver seconded. Mr. Marks stated that the present site was estimated to be worth from £30,000 to £35,000, and if sold the proceeds would enable the committee to build a new hospital, and to endow the hospital with a substantial sum as well. They had obtained a convenient site in Hunter Street, and, as it was important there should be no further delay, if the court authorised the resolution the committee would proceed at once. Mr. H. A. Reeves, member of the committee, and one of the honorary surgeons, strongly objected to the proposal, and suggested an alternative scheme for rebuilding on the present site, which he supported at length. He thought the committee had not fully taken the governors and supporters of the charity into their confidence, and that some means ought to be taken to obtain their views on the rival schemes. After a few words from Mr. Flower, M.P., the resolution was carried with two dissentients, and a vote of thanks to the chairman concluded the proceedings.

### OPEN-AIR BALCONIES AT THE NORTH LONDON HOSPITAL FOR CONSUMPTION.

Last week (June 20th) the new open-air balconies which have been added to the North London Hospital for Consumption, Hampstead, were formally opened by Sir Henry Harben, the occasion being celebrated by a very pleasant and well-attended "garden party." The open-air treatment has been in vogue at the hospital for about a year, with most satisfactory results. Situated high above London smoke and fog, with ample grounds and plenty of breathing space, the hospital lends itself well to such a purpose, and it is encouraging in the extreme to those who are fighting one of the most deadly of diseases that the treatment which yields such good results can be successfully carried out not only in England, but in London itself. At first two wards were set apart for "open-air cases," then all those on the south-west side of the building were utilised, and now a specially designed wing has been built, which will accommodate forty beds. Here patients will practically live, day and night, in the fresh air, shielded from too much wind or sun, and enabled to get all the benefit possible from the treatment. Th balconies are pleasant places; the walls (what there is of them) are tiled half way up, the rest distempered, the floors are covered with linoleum, and the open side, facing south-west, is shaded by green sun-blinds. They were bright with hanging baskets and pots of flowers, and immediately after the opening day were ready for occupation. The cost of this structure has been £3,000, and it has been so planned as to form part of the final wing of the hospital, for which the cost is estimated at £8,000, towards which the committee are earnestly asking for funds.

People who are afraid of "draughts" should be invited to visit the North London Hospital, where, it is safe to say, their nerves would receive a somewhat severe shock. Doors, windows, ventilators, all are open wide day and night; but so far from objecting to being sometimes nearly blown out of bed, the patients seem to revel in the new system, and their bright looks and the tales of improvement on all sides speak for themselves.

The report for the past year is extremely interesting, a comparison of 168 cases under the old and new system showing a wonderful increase in the percentage of those "much improved" and "improved." In carrying out this treatment the committee have considerably extended the length of time patients can stay in the hospital, a step which much enhances their chance of real recovery; and here is the need sorely felt for a convalescent home, where patients may be sent before returning to their homes. A study of the expenditure tables for the year shows that while an increase in the sum spent on "provisions" is consequent on the admission of more patients, and the very liberal diet which forms part of the open-air treatment, yet the cost per occupied bed is slightly lower than in the previous year, a fact which speaks well for the economy of management.

### ALEXANDRA HOSPITAL FOR CHILDREN WITH HIP DISEASE.

The thirty-third annual meeting of the Alexandra Hospital for Children with Hip Disease, Bloomsbury, was held at the hospital on Thursday, the 21st inst., under the presidency of Mr. W. H. Whitfield, chairman of the committee.

The report and accounts for the year ended September 30th, 1899, submitted by the committee of management, showed that the total ordinary income was £3,678. The ordinary expenditure amounted to £2,194, and the extraordinary—being an amount set aside to meet expenses in connection with the building fund—to £1,300, a total expenditure of £3,494. There is consequently a small balance in hand of £384, which the committee state they have been able to show owing to the support extended during the past financial year, though it is to some extent accounted for by the fact that in consequence of the rebuilding operations the number of beds available has been necessarily less than when the hospital is in full working order. The committee congratulate their supporters on the fact that the annual subscriptions received, namely £1,238, showed the considerable increase of £219 on those for the previous period, while the donations, at £1,260, also showed an improvement of £10. The annual subscriptions for the maintenance of special cots, £711, showed, however, a decrease of £8. There were among the donations received during the year one of £300 from Miss E. W. Henriques, and another of £250 from Messrs. Barnato Bros., as executors of the late Woolf Joel. As regards the building fund, the committee have the pleasure to inform their supporters that the new hospital, which was opened by the Princess of Wales—who was accompanied by the Prince of Wales and the Princess Victoria—on July 20th, 1899, is now in working order. The total cost has been about £20,000. A festival dinner in connection with the building fund, at which the Duke of Cambridge presided, was held at the Hotel Cecil on June 20th, 1899, when upwards of £4,000 was obtained. The only debt now remaining in connexion with the building fund is a sum of about £250, which is required to complete the furnishing. The receipts on this account during the financial year, including £1,631 from Mr. and Mrs. Henry Partridge in three separate amounts, and £500 respectively from the Prince of Wales's Hospital Fund and "H. D. B." were £6,362. The expenditure was £10,192, leaving an adverse balance of £3,830 to be carried to the balance-sheet, where the building fund capital account, which stood at £7,403 on September 30th, 1898, is consequently reduced to £3,573. Of the 68 beds—60 in London and 8 at the convalescent home at Painswick—only a small number of the former were available owing to the rebuilding operations previously referred to. During the year 65 children were under treatment, and of these 8 were discharged cured, 15 were improved, 1 was transferred to a general hospital, 16 were sent to convalescent homes, and 25 remained under treatment on September 30th. The number of individual out-patients during the year was 350, and the number of attendances—including the visits paid by the out-patient nurse to children at their own homes—was 2,212. During the year Mr. Henry Partridge and Dr. H. Willingham Gill have been added to the committee.

The adoption of the report, which had been previously circulated, was moved by the Chairman, who said it was the most satisfactory one since the hospital was started, at any rate in the fifteen years he had been connected with it. Major Hastings Brooke seconded the motion, which was carried unanimously.

The retiring members of the committee, trustees, medical officers, auditors, and treasurer were re-elected, and the usual votes of thanks to the medical and surgical staff, the visiting ladies, and to the chairman for his services concluded the business of the meeting.

### THE BELGRAVE HOSPITAL FOR CHILDREN.—LAYING THE FOUNDATION STONE.

On Wednesday afternoon the Princess Henry of Battenberg laid the foundation stone of the new Belgrave Hospital for Children, Clapham Road, Kennington. Her Royal Highness arrived at the site at four o'clock, and was met by Lord Windsor, the Executive Committee, the Bishop of Rochester, the Vicar of Kennington, and the Members of Parliament for the district. The proceedings were opened by the presentation of a basket of flowers by four young ladies, after which Lord Windsor gave a short address, touching upon the salient points of the committee's intentions concerning the new hospital. He said that the needs of South London for hospital accommodation for children exceeded even that of North London, and the new building was intended only for those who were not able to provide medical skill or sick comforts for themselves. He promised that the greatest care should be taken to exclude from the benefits of the charity those who could afford to pay for ordinary medical attention on their children. It was unnecessary on this occasion to draw comparisons between those hospitals which accept payments and those which do not, but, after mature consideration of both methods by the committee, the latter had been selected as the most suitable. There was the greatest necessity to provide for those children who could not provide against their sickness and adversity. A sum of £50,000 was required in order to erect the new hospital, of which £17,000 had already been subscribed. The cost of the site was £4,000; of the centre block and west wing, £25,000; of the out-patient department, £5,000; of the east wing £8,000, and of the south wing £6,000. The centre block would provide the offices of administration, and would be large enough for the completed hospital. He earnestly entreated the support of the public, and solicited gifts in kind. One generous donor had promised the fittings of the operating theatre, and they would be of that complete and perfect character demanded by modern surgery. He could not forbear to mention the encouragement and assistance which had been given to their project by the late Duke of Westminster, and which was still extended to them by the present Duke. In conclusion, he offered the respectful and grateful thanks of the committee to the Princess for the assistance and encouragement she had given them by her presence. The Architect then presented Her Royal Highness with an inscribed silver trowel, and the Bishop of Rochester conducted an appropriate service. At the appointed place the Princess laid the stone, with the following words: "In the faith of Jesus Christ we place this foundation-stone, in the name of the Father, and of the Son, and of the Holy Ghost." At the conclusion of the religious ceremony Lord Windsor presented the members of the committee to their Royal guests, and afterwards about thirty purses containing from £5 5s. to £40 apiece were given by children. Mr. F. L. Cook, M.P., proposed the vote of thanks to the Princess, and Sir Robert Mowbray, Bart., M.P., seconded it. The music throughout was provided by the children's orchestra, who acquitted themselves of their task prettily and effectively.

